# Pregnancy Outcomes After Second Trimester Pregnancy Loss and Termination for Medical Reasons Before 24 Weeks: A Historical Cohort Study [PASTeL‐2]

**DOI:** 10.1111/1471-0528.70161

**Published:** 2026-01-25

**Authors:** Andrea M. F. Woolner, Konstantin Shestopaloff, Alexander E. P. Heazell

**Affiliations:** ^1^ School of Medicine, Institute of Applied Health Sciences, Medical Sciences and Nutrition, Aberdeen Centre for Women's Health Research, University of Aberdeen Aberdeen UK; ^2^ Institute of Applied Health Sciences, University of Aberdeen Aberdeen UK; ^3^ Faculty of Biology, Medicine and Health School of Medical Science, Tommy's Maternal and Fetal Health Research Centre, University of Manchester Manchester UK; ^4^ Saint Mary's Hospital, Manchester University NHS Foundation Trust Manchester UK

**Keywords:** late miscarriage, late spontaneous abortion, midtrimester miscarriage, spontaneous preterm birth, termination for fetal anomalies, termination for medical reason

## Abstract

**Objective:**

To investigate if second trimester pregnancy loss (second trimester miscarriage [STM] or termination for medical reasons [TFMR]) was associated with subsequent adverse pregnancy outcomes.

**Design:**

Retrospective cohort study.

**Setting:**

Conducted using the Aberdeen Maternity and Neonatal Databank [AMND] in Aberdeen, United Kingdom.

**Population:**

Women with and without a history of STM or TFMR (between 13 + 0 and 23 + 6 weeks' gestation).

**Methods:**

Logistic and linear regression were used to determine associations between exposed (prior STM or TFMR) and unexposed women (women with prior livebirth).

**Main Outcome Measures:**

The primary outcome was subsequent spontaneous preterm birth, defined as spontaneous onset of labour and birth between 24 + 0 and 36 + 6 weeks' gestation.

**Results:**

The study included 65 592 women with first and second pregnancies recorded from 1950 to 2017. Women who had a STM in their first pregnancy (*n* = 935) were at significantly greater risk of spontaneous preterm birth in the next pregnancy (4.3% vs. 1.5%; adjusted Odds Ratio [aOR] 2.55 (95% CI 1.81 to 3.50); *p* < 0.01). Women with STM in their first pregnancy were two‐fold more likely to have a repeat second trimester miscarriage (3.7% vs. 1.1%; aOR 2.25 (95% CI 1.53 to 3.19); *p* < 0.01). Women who had a first TFMR (*n* = 177) were significantly more likely to have a repeat TFMR (adjusted OR [aOR] 6.59 (3.4% vs. 0.3%, 95% CI 2.54 to 13.99); *p* < 0.01). There was no observed increased risk of spontaneous preterm birth after TFMR detected in this sample (aOR 1.06 (95% CI 0.39 to 2.87); *p* = 0.91) though the sample size was too small to be conclusive.

**Conclusions:**

Women with a history of second trimester pregnancy loss have an increased risk of adverse pregnancy outcomes in a subsequent pregnancy. Consequently, antenatal care surveillance and counselling may need to be increased for women with a prior STM, who are at risk of spontaneous preterm birth and other adverse obstetric outcomes including pre‐eclampsia. Women after TFMR can be reassured by our findings; however, larger cohorts are needed to confirm these results.

## 1 Introduction

Second trimester miscarriage (STM) occurs in approximately 0.5%–1% of pregnancies [1, 2, 3]. STM is defined as a spontaneous pregnancy loss which occurs from 13 to 23 + 6 weeks' gestation, though the definition varies [4]. Over 4000 pregnancies per year are terminated for medical reasons (TFMR) most often for fetal anomalies in the UK. The majority of TFMR occur in the second trimester [5]. Many couples fear what impact their loss will have on their next pregnancy, and what their risk of recurrent loss is. There are no published international or UK guidelines dedicated to the management of STM or TFMR; specifically, no guidance on what, if any, changes should be made to subsequent antenatal care.

Research suggests that other types of pregnancy loss, specifically recurrent first trimester miscarriages [6] and stillbirth [7, 8] adversely affect subsequent pregnancies with a greater risk of repeat loss and preterm birth. We hypothesised that having a STM or TFMR could increase the risk of subsequent adverse pregnancy events including spontaneous preterm birth either due to the premature birth affecting cervical integrity or due to the pathophysiology involved in the second trimester loss itself such as placental disorder, a propensity to chronic infection or inflammation, and in the case of both STM and TFMR an increased risk associated with the presence of chromosomal aneuploidy. Conversely, many second trimester losses are unexplained [9] or are due to factors such as acute infection which may be less likely to recur. Placental dysfunction is known to recur in subsequent pregnancies thus it could be implicated in pregnancies after a STM [10]. Our systematic review [11] However, literature was scarce, studies were of low quality and none had delineated spontaneous preterm birth from iatrogenic preterm births [11]. Critically, no studies had considered potential confounding factors in their analyses [11]. Evidence on the impact of induced abortion in the second trimester is conflicting [12, 13, 14, 15, 16, 17, 18, 19]. We found no studies which had specifically investigated TFMR [11].

The Aberdeen Maternity Neonatal Databank (AMND) [20] provides an opportunity to study subsequent pregnancies in a detailed, validated, historical cohort from Aberdeen, UK with a low outmigration rate. The objective of this study was to determine if women with a second trimester pregnancy loss [defined as (i) second trimester spontaneous miscarriage from 13 + 0 to 23 + 6 weeks' gestation (ii) TFMR 13 + 0 to 23 + 6 weeks' gestation] were at increased risk of spontaneous preterm birth and other adverse pregnancy outcomes in subsequent pregnancies.

## 2 Methods

A cohort study was conducted using routinely collected hospital data for women who had pregnancies recorded within Aberdeen Maternity Hospital, UK using the historical database, Aberdeen Maternity and Neonatal Databank (AMND) [20] which is a validated data source. This study set out to answer the four research questions below:

1. Does an initial second trimester miscarriage lead to an increased risk of adverse pregnancy outcomes in the second pregnancy?
2. Does a second trimester miscarriage in any prior pregnancy lead to an increased risk of adverse pregnancy outcomes in subsequent pregnancies?
3. Does an initial second trimester TFMR/FA lead to an increased risk of adverse pregnancy outcomes in the second pregnancy?
4. Does a second trimester TFMR/FA in any pregnancy lead to an increased risk of adverse pregnancy outcomes in subsequent pregnancies?

The population included women who had at least two singleton pregnancies recorded within the AMND from 1950 to 2017. The exposures for each research question were as follows: (i) a miscarriage (spontaneous/missed miscarriage) between 13 + 0 and 23 + 6 weeks' gestation in the first pregnancy (ii) a miscarriage (spontaneous/missed miscarriage) between 13 + 0 and 23 + 6 weeks' gestation in the first or any subsequent pregnancy (iii) a TFMR in the first pregnancy between 13 + 0 and 23 + 6 weeks' gestation (iv) a TFMR between 13 + 0 and 23 + 6 weeks' gestation in any of the first four pregnancies. The unexposed cohort included all women with at least two singleton pregnancies recorded within the AMND, with a first livebirth. Women who had an initial pregnancy which ended in stillbirth, first trimester miscarriage, ectopic pregnancy, molar pregnancy or induced abortion in their first pregnancy were excluded; in the case of stillbirth this is because high‐quality data [21] have established increased risk of subsequent pregnancy complications. Women with multiple pregnancies were also excluded. The primary outcome for all research questions was spontaneous preterm birth, defined as spontaneous onset of labour and birth between 24 + 0 and 36 + 6 weeks' gestation. Secondary outcomes for research questions 1 and 2 included: late miscarriage [defined as spontaneous/missed abortion between 13 + 0 and 23 + 6 weeks' gestation]; stillbirth; early miscarriage [spontaneous/missed miscarriage < 13 weeks' gestation]; pre‐eclampsia; antepartum haemorrhage (APH); second birth outcomes (any); neonatal unit (NNU) admission; mode of second birth [assisted breech, caesarean section (CS), forceps, rotational forceps, spontaneous vaginal birth (SVD), vacuum delivery]; labour type [spontaneous, elective CS, induction of labour]; birthweight; crown‐rump length (CRL); sex of baby. Secondary outcomes for research questions 3 and 4 were: repeat TFMR; early miscarriage; APH; NNU admission; mode of birth and labour type. We reviewed the published core outcome set for miscarriage trials and reviews [22], in deciding upon on our outcomes, however many were not relevant to research on subsequent pregnancy outcomes following miscarriage.

### 2.1 Definitions of Outcomes

Gestation at birth is coded according to the due date that was estimated by the first trimester ultrasound scan when available from hospital records (from 1986 onwards) and otherwise by the last menstrual period date that was recorded at first antenatal booking and the date of birth. Antepartum haemorrhage (APH) was defined in the AMND as vaginal bleeding after 24 weeks' gestation which includes abruption and placenta praevia and a binary variable APH yes/no was computed for this study. Pre‐eclampsia is defined as gestational hypertension and at least 1 episode of proteinuria (0.3 g protein in 24 h); this information was directly collected from the hospital records.

### 2.2 Definition of Confounders

Deprivation was recorded using Scottish index of multiple deprivation (SIMD 2016) [23], where 1 is the least deprived and 10 is the most deprived and this was recorded routinely in the AMND. SIMD [23] is a marker of socioeconomic status in Scotland and is an objective measure of how deprived that area is, and uses information from seven categories such as income, employment, education, health access to services, crime, and housing. Maternal age at baby's birth was collected routinely by the AMND from the hospital medical records. Smoking status was self‐reported at the time of antenatal booking and documented within the hospital record from which it was collected for inclusion within the AMND.

### 2.3 Permissions and Data Source

This study is reported in accordance with the STROBE guidance [24]. Approval was obtained from the AMND steering committee (reference: AMND2023‐01) to undertake this study. The AMND steering committee has overarching ethical approval for studies which use pseudo‐anonymised data with no data linkage and therefore formal ethics approval was not required. The AMND holds routinely collected pregnancy data for all women who gave birth in Aberdeen Maternity Hospital from 1949 until 2017 [20]. All pregnancy records were included automatically in the AMND until 2017 (when data collection to this resource ceased) [20], and the information was entered routinely for all women under the jurisdiction of Aberdeen Maternity Hospital until 2017, which is the only maternity hospital in the area. A pseudo‐anonymised dataset was provided to the researchers and was analysed within the Grampian data safe haven in accordance with UK data protection laws. Data can be made available by applying to the AMND for permission (amnd@abdn.ac.uk).

### 2.4 Statistical Analyses

*For research questions 1 and 3*, analysis was conducted using logistic regression for the primary outcome and for all of the secondary outcomes except birthweight, CRL and gestation which were analysed using linear regression. The model was adjusted for demographic factors: age ([16,25), [25,35), [35+]), BMI ([0, 20), [20, 25), [25, 30), [30+], NA), smoking ([Smoker], [Ex‐Smoker], [Non‐Smoker], [not available {NA}]) and SIMD ([0, 5], [5+], NA). Subjects with missing age were excluded from the analysis and BMI, Smoking and SIMD included a separate NA (missing) category, due to high missingness. No multiple comparison adjustment was made for the primary outcome. Multiple comparison adjustments were set to 13 for secondary outcomes, giving *p*‐value cutoffs of 0.05 and 0.05/13, respectively. *For research question 2*, a total of three scenarios were considered: (i) adverse event in first pregnancy affecting the second or subsequent pregnancy, (ii) adverse event in the first or the second pregnancy affecting the third or subsequent pregnancies; (iii) adverse event in the first, second or third pregnancy affecting the fourth or subsequent pregnancies. Analysis was conducted using logistic regression for the primary outcome and three secondary outcomes (adverse event [miscarriage, induced abortion or stillbirth], late miscarriage and first trimester miscarriage). The model was adjusted for demographic factors: age ([16,25), [25,35), [35+]), BMI ([0, 20), [20, 25), [25, 30), [30+], NA), smoking (Smoker, Ex‐Smoker, Non‐Smoker, NA) and SIMD ([0, 5], 5+, NA), as well as three risk factors: pre‐eclampsia, APH and stillbirth, having occurred in any previous pregnancy. Subjects with missing age were excluded from the analysis. Smoking, BMI and SIMD were imputed using carry forward of the previous value and the first observation was imputed using carry backward from the first available value. As this still resulted in a high number of missing, a separate missing category was included for these three variables. Missing risk factors variables were set to zero (absent). Multiple comparison adjustments were set to 3 for the main outcomes and 12 for secondary outcomes, giving *p*‐value cutoffs of 0.05/3 and 0.05/12, respectively. *For research question 3*, analysis was carried out using logistic regression for all but birthweight, CRL and gestation which were analysed using linear regression. The model was adjusted for demographic factors: age ([16,25), [25,35), [35+]), BMI ([0, 20), [20, 25), [25, 30), [30+]), smoking (Smoker, Ex‐Smoker, Non‐Smoker) and SIMD ([0, 5], 5+). Subjects with missing demographic information were excluded from the analysis. No multiple comparison adjustment was made for the primary outcome. Multiple comparison adjustments were set to 11 for secondary outcomes, giving *p*‐value cutoffs of 0.05 and 0.05/11, respectively. *For research question 4*, three scenarios were included in a logistic regression model: (i) adverse event in first pregnancy affecting second or subsequent, (ii) adverse event in first or second, affecting third or subsequent and (iii) adverse event in first, second or third affecting fourth or subsequent pregnancies. The model was adjusted for demographic factors: age ([16,25), [25,35), [35+]), BMI ([0, 20), [20, 25), [25, 30), [30+], NA), smoking (Smoker, Ex‐Smoker, Non‐Smoker, NA) and SIMD ([0, 5], 5+, NA), as well as three risk factors: pre‐eclampsia, APH and stillbirth, having occurred in any previous pregnancy. Subjects with missing age were excluded from the analysis. Smoking, BMI and SIMD were imputed using carry forward of the previous value and the first observation was imputed using carry backward from the first available value. As this still resulted in a high number of missing, a separate missing category was included for these three variables. Missing risk factors variables were set to zero (absent). Multiple comparison adjustments were set to 3 for main outcomes and 12 for the secondary outcomes, giving *p*‐value cutoffs of 0.05/3 and 0.05/12, respectively. For all analyses, Odds Ratios unadjusted (uOR) and adjusted (aOR) were presented with 95% confidence intervals (95% CI). Nominal statistical significance was defined as *p* < 0.05 with Bonferroni adjusted cutoffs determined by the number of multiple comparisons.

## 3 Results

### 3.1 Second Trimester Miscarriage in the First Pregnancy

935 women had a STM in their first pregnancy. Table 1 contains the demographic and second pregnancy characteristics for women who had either a STM or livebirth in their first pregnancy. Table 2 shows the comparative analyses for second pregnancy outcomes. Women who had a first pregnancy which ended in STM were significantly more likely to have spontaneous preterm birth (4.3% vs. 1.5%; aOR 2.55 [95% CI 1.81 to 3.50] *p* < 0.01) in their second pregnancy. Similarly, the risk of STM was higher in the second pregnancy where women had an initial STM (3.7% vs. 1.1%; aOR 2.25 [95% CI 1.53 to 3.19] *p* < 0.01). Women who had an initial STM were also more likely to have an early miscarriage (8.7% vs. 5.0%; aOR 1.44 [95% CI 1.13 to 1.82] *p* < 0.01). Women with a first STM were at more than twice the odds of having pre‐eclampsia in their second pregnancy (23.4% vs. 11.7%; aOR 2.24 [95% CI 1.91 to 2.61]; *p* < 0.01). Women who had a first STM had second babies weighing 243 g lighter (95% CI 205 to 281 g, *p* < 0.01) than women with a first livebirth, noting that women with a prior STM had 6.4% second births born at preterm gestations compared to 2.3% of women with a prior LB. There was no significant difference when maternal ethnicity was included in the model for birthweight. Women who had a first late miscarriage had second babies with a CRL at their 12‐week ultrasound scan of 1.8 mm less than babies born to women with a first livebirth (95% CI 1.13 to 2.52 mm, *p* < 0.01). There was no significant difference when maternal ethnicity was included in the model. There was no difference in the sex of babies born in second pregnancies after a late miscarriage or in women with a previous livebirth (*p* = 0.80).

**TABLE 1:** Demographic and second pregnancy characteristics of women with a first second trimester miscarriage compared to women with a first livebirth.

Demographic characteristic	All women (*N* = 65 592)	Women with 1st late miscarriage (*N* = 935) *n* (%)	Women with 1st livebirth (*N* = 64 657) *n* (%)	*p*
Age				
16–25	22 452	448 (47.9)	22 004 (34.0)	< 0.01
25–35	37 451	405 (43.3)	37 046 (57.3)	
36+	5661	82 (8.8)	5579 (8.7)	
Missing	28	0	28	
BMI				
< = 20	5981	57 (6.1)	5924 (9.2)	0.01
20–25	30 927	245 (26.2)	30 628 (47.4)	
26–30	13 999	136 (14.5)	13 863 (21.4)	
> 30	6353	75 (8.0)	6278 (9.7)	
Missing	8332	422 (45.1)	7910 (12.2)	
Diabetes				
No	64 743	927 (99.1)	63 816 (98.7)	0.3
Yes	849	8 (0.9)	841 (1.3)	
Smoking status				
Non smoker	34 432	414 (44.3)	34 018 (52.6)	< 0.01
Ex smoker	1790	25 (2.7)	1765 (2.7)	
Smoker	14 834	258 (27.6)	14 576 (22.5)	
Missing	14 536	238 (25.5)	14 298 (22.1)	
SIMD				
1–5	15 298	233 (24.9)	15 065 (23.3)	< 0.01
6–10	30 357	363 (38.8)	29 994 (46.4)	
Missing	19 937	339 (36.3)	19 598 (30.3)	
Ethnicity				
Black/Asian/MENA	1176	18 (1.9)	1158 (1.8)	0.623
White	36 461	486 (52.0)	35 975 (55.6)	
Missing	27 955	431 (46.1)	27 524 (42.6)	
Maternal pre‐existing hypertension				
No	57 768	716 (76.6)	57 052 (88.2)	< 0.01
Yes	7824	219 (23.4)	7605 (11.8)	
Gestation at second birth				
0–23	7241	141 (15.1)	7100 (11.0)	< 0.01
24–36	1527	60 (6.4)	1467 (2.3)	
37+	54 206	692 (74.0)	53 514 (82.8)	
Missing	2618	42 (4.5)	2576 (4.0)	
Amniocentesis in second pregnancy				
No	64 412	914 (97.8)	63 498 (98.2)	0.36
Yes	1180	21 (2.2)	1159 (1.8)	
TFMR in second pregnancy				
Yes	2825	17 (1.8)	2808 (4.3)	0.01
No	62 767	918 (98.2)	61 849 (95.7)	
Decade of birth				
1950	1486	5 (0.5)	1481 (2.3)	< 0.01
1960	8812	107 (11.4)	8705 (13.5)	
1970	7019	150 (16.0)	6869 (10.6)	
1980	10 764	202 (21.6)	10 562 (16.3)	
1990	12 129	128 (13.7)	12 001 (18.6)	
2000	9475	158 (16.9)	9317 (14.4)	
2010	8031	91 (9.7)	7940 (12.3)	
2020	2852	15 (1.6)	2837 (4.4)	
Missing	5024	79 (8.4)	4945 (7.6)	
Sex of baby				
Male	29 000	394 (42.1)	28 606 (44.2)	0.80
Female	27 615	383 (41.0)	27 232 (42.1)	
Missing	8977	158 (16.9)	8819 (13.6)	

**TABLE 2:** Comparison of second pregnancy outcomes for women whose first pregnancy resulted in late miscarriage versus those for women whose first pregnancy resulted in livebirth.

Second pregnancy outcome	All women	Women with 1st LM (*N* = 935)	Women with 1st LB (*N* = 64 657)	Unadjusted OR	95% CI	*p*	Adjusted OR^a^	95% CI	*p*
Late miscarriage (*N* = 65 592, all second pregnancy outcomes)									
No	64 862	902 (96.5)	63 960 (98.9)	3.36	2.31 to 4.08	< 0.01	2.25	1.53 to 3.19	< 0.01
Yes	730	33 (3.7)	697 (1.1)						
Spontaneous preterm birth									
No	62 113	858 (91.8)	61 255 (94.7)	2.58	2.03 to 3.89	< 0.01	2.55	1.81 to 3.50	< 0.01
Yes	1041	40 (4.3)	1001 (1.5)						
Missing/not applicable	2438	37 (4.0)	2401 (3.7)						
Stillbirth									
Yes	352	9 (1.0)	343 (0.5)	1.92	0.92 to 3.52	0.05	1.78	0.84 to 3.29	0.09
Early miscarriage									
No	56 205	756 (80.9)	55 449 (85.8)	1.85	1.45 to 2.31	< 0.01	1.44	1.13 to 1.82	< 0.01
Yes	3301	81 (8.7)	3220 (5.0)						
Other birth outcomes (not miscarriage or LB)—not included in analysis	6086	98 (10.5)	5988 (9.3)						
Pre‐eclampsia									
No	57 798	716 (76.6)	57 082 (88.3)	2.30	1.97 to 2.68	< 0.01	2.24	1.91 to 2.61	< 0.01
Yes	7794	219 (23.4)	7575 (11.7)						
Antepartum haemorrhage (APH)									
No	61 984	879 (94.0)	61 105 (94.5)	1.10	0.83 to 1.42	0.51	1.16	0.87 to 1.51	0.29
Yes	3608	56 (6.0)	3552 (5.5)						
Subsequent adverse composite outcome (including ectopic pregnancy/M pregnancy/induced abortion/early miscarriage/stillbirth) [all compared to second pregnancy = livebirth]									
Ectopic/molar	369	8 (0.9)	361 (0.6)	0.70	0.59 to 0.82	< 0.01	0.94	0.79 to 1.12	0.47
Induced abortion	3936	32 (3.4)	3904 (6.0)						
Livebirth	56 205	756 (80.9)	55 449 (85.8)						
Early miscarriage	4651	130 (13.9)	4521 (7.0)						
Stillbirth	352	9 (1.0)	343 (0.5)						
Missing/not applicable	79	0	79 (0.1)						
NNU admission									
No	62 803	874 (93.5)	61 929 (95.8)	2.03	1.51 to 2.68	< 0.01	1.86	1.38 to 2.47	< 0.01
Yes	2789	61 (6.5)	2728 (4.2)						
Mode of birth									
Assisted Breech	590	14 (1.5)	576 (0.9)	3.33	2.88 to 3.84	< 0.01	4.19	3.60 to 4.88	< 0.01
Caesarean Section	7690	132 (14.1)	7558 (11.7)						
Forceps	2099	150 (16.0)	1949 (3.0)						
Kiellands Forceps	325	14 (1.5)	311 (0.5)						
SVD	45 025	421 (45.0)	44 604 (69.0)						
Vacuum	797	40 (4.3)	757 (1.2)						
Missing/not applicable	9066	164 (17.5)	8902 (13.8)						
Elective CS	4533	36 (3.9)	4497 (7.0)	1.44	1.24 to 1.66	< 0.01	1.40	1.21 to 1.62	< 0.01
Induced	13 048	270 (28.9)	12 778 (19.8)						
Spontaneous	39 054	475 (50.8)	38 579 (59.7)						
Missing/not applicable	8957	154 (16.5)	8803 (13.6)						

*Note:* Some variables include ‘missing/not applicable’ because some women had second pregnancies which ended before 24 weeks.

^a^ Adjusted for smoking, maternal age, BMI and SIMD (deprivation).

### 3.2 Second Trimester Miscarriage in Any Pregnancy

Subsequent pregnancy outcomes for women who had a STM in any of their prior pregnancies were compared to subsequent pregnancy outcomes for women who had only live births in up to their first four pregnancies (results shown in Tables S1, S2 and 3). Having a STM in any previous pregnancy conveys more than a 1.5 increased odds of subsequent spontaneous preterm birth (Table 3). Women who had a STM in any prior pregnancy overall appeared to have an increased risk of repeat STM and early miscarriage.

**TABLE 3:** Odds Ratios comparing subsequent adverse pregnancy outcomes in women with and without STM in any prior pregnancy.

Subsequent pregnancy outcomes	Unadjusted OR	95% CI	*p*	Adjusted OR^a^	95% CI	*p*
Subsequent late miscarriage						
For women with 2+ pregnancies, where event in first pregnancy	2.28	1.79 to 3.18	< 0.01	1.54	1.15 to 2.06	0.004
For women with 3+ pregnancies, where event in first or second pregnancy	1.47	1.11 to 1.95	0.0067	1.24	0.93 to 1.65	0.14
For women with 4+ pregnancies, where event in first, second or third pregnancy	2.04	1.53 to 2.72	< 0.01	1.89	1.41 to 2.54	< 0.01
Subsequent first trimester miscarriage						
For women with 2+ pregnancies, where event in first pregnancy	2.379	2.00 to 2.83	< 0.01	2.13	1.78 to 2.54	< 0.0001
For women with 3+ pregnancies, where event in first or second pregnancy	1.4472	1.23 to 1.71	< 0.01	1.38	1.17 to 1.63	0.0002
For women with 4+ pregnancies, where event in first, second or third pregnancy	1.1444	0.94 to 1.40	0.19	1.09	0.89 to 1.34	0.4006
Subsequent spontaneous preterm birth						
For women with 2+ pregnancies, where event in first pregnancy	2.7159	2.08 to 3.55	< 0.01	2.2931	1.74 to 3.02	< 0.01
For women with 3+ pregnancies, where event in first or second pregnancy	1.7304	1.32 to 2.27	< 0.01	1.6529	1.25 to 2.18	< 0.0004
For women with 4+ pregnancies, where event in first, second or third pregnancy	1.9287	1.42 to 2.61	< 0.01	1.9368	1.42 to 2.64	< 0.01
Subsequent adverse composite outcome (including ectopic pregnancy/molar pregnancy/induced abortion/early miscarriage/stillbirth) [all compared to second pregnancy = livebirth]						
For women with 2+ pregnancies, where event in first pregnancy	1.5733	1.37 to 1.81	< 0.0001	1.2948	1.12 to 1.50	0.0006
For women with 3+ pregnancies, where event in first or second pregnancy	0.8216	0.73 to 0.93	0.0017	0.765	0.67 to 0.87	< 0.01
For women with 4+ pregnancies, where event in first, second or third pregnancy	0.6863	0.60 to 0.79	< 0.0001	0.6431	0.56 to 0.74	< 0.01

^a^ Adjusted for smoking, maternal age, BMI and SIMD (deprivation).

### 3.3 TFMR in the First Pregnancy

177 women had a TFMR in their first pregnancy. Demographic and subsequent pregnancy characteristics for women with and without a TFMR in their first pregnancy are shown in Tables S3 and S4. Comparative results for women with and without a history of first TFMR are shown in Tables 4 and 5. There was no statistically significant difference in the proportions of spontaneous preterm birth between women who had a first TFMR compared to women who had a first livebirth; however, due to low counts, the raw data cannot be shown. This result should be interpreted with caution given the small sample size. Women who had a first TFMR were six times more likely to have a repeat TFMR in their second pregnancy than women who had a first livebirth (3.4% vs. 0.3%; aOR 6.59 [95% CI 2.55 to 13.99] *p* < 0.01). There was no associated increase in early miscarriage, pre‐eclampsia, or antepartum haemorrhage after a first TFMR. There was no statistically significant difference in the birthweights of second babies born to women who had a first TFMR compared to women who had a first livebirth (*p* = 0.93). There was no statistically significant difference in the first trimester crown‐rump lengths of babies born to women who had a first TFMR compared to women who had a first livebirth (*p* = 0.60).

**TABLE 4:** Comparison of second pregnancy outcomes for women whose first pregnancy resulted in TFMR versus those for women whose first pregnancy resulted in livebirth.

Second pregnancy outcome	All women	Women with 1st TFMR (*N* = 177)	Women with 1st LB (*N* = 64 954)	Unadjusted OR	95% CI	*p*	Adjusted OR^a^	95% CI	*p*
TFMR in second pregnancy									
Yes	194	6 (3.4)	188 (0.3)	12.07	4.70 to 25.33	< 0.01	6.59	2.55 to 13.99	< 0.01
Early miscarriage									
No	51 570	133 (75.1)	51 437 (79.2)	1.92	1.08 to 3.17	0.017	1.31	0.73 to 2.18	0.3286
Yes	3039	15 (8.5)	3024 (4.7)						
Other birth outcomes (not miscarriage or LB)—not included in analysis	10 345	29 (16.4)	10 316 (15.9)						
Antepartum haemorrhage (APH)									
No	56 862	149 (84.2)	56 713 (87.3)	1.77	1.00 to 2.91	0.04	1.44	0.81 to 2.38	0.18
Yes	3238	15 (8.5)	3223 (5.0)						
Missing	4854	13 (7.3)	4841 (7.5)						
NNU admission^b^									
No	18 430	86 (20.3)	18 344 (28.2)	2.59	1.68 to 3.88	< 0.01	2.46	1.58 to 3.72	< 0.01
Yes	2502	30 (22.6)	2472 (3.8)						
Missing	44 022	61 (34.5)	43 961 (67.7)						
Mode of birth^b^									
Any Forceps/breech extraction/ventouse	3372	36 (20.3)	281 (0.4)	5.40	3.83 to 7.64	< 0.01	3.92	2.76 to 5.61	< 0.01
Caesarean Section	6955	40 (22.6)	6915 (10.6)						
SVD	41 540	57 (32.2)	41 483 (63.9)						
NA	13 087	44 (24.9)	13 043 (20.1)						
Labour type^b^									
Elective CS	4113	9 (5.1)	4104 (6.3)	1.44	1.06 to 1.95	0.0207	1.19	0.87 to 1.63	0.2641
Induced	11 723	40 (22.6)	11 683 (18.0)						
Spontaneous	36 123	84 (47.5)	36 039 (55.5)						
Missing	4113	9 (5.1)	4104 (6.3)						

^a^ Adjusted for smoking, maternal age, BMI and SIMD (deprivation).

^b^ Not all women included where second pregnancy ended < 24 weeks.

**TABLE 5:** Odds Ratios comparing subsequent adverse pregnancy outcomes in women with and without TFMR in any prior pregnancy.

Subsequent pregnancy outcomes	Unadjusted OR	95% CI	*p*	Adjusted OR^a^	95% CI	*p*
Subsequent TFMR						
For women with 2+ pregnancies, where event in first pregnancy	9.35	4.75 to 18.43	< 0.0001	5.71	2.88 to 11.33	< 0.01
For women with 3+ pregnancies, where event in first or second pregnancy	5.36	2.61 to 11.03	< 0.0001	3.14	1.52 to 6.49	0.0021
For women with 4+ pregnancies, where event in first, second or third pregnancy	5.09	2.02 to 12.82	0.0006	2.62	1.03 to 6.69	0.0434
Subsequent first trimester miscarriage						
For women with 2+ pregnancies, where event in first pregnancy	1.95	1.28 to 2.96	0.0017	1.65	1.08 to 2.50	0.0199
For women with 3+ pregnancies, where event in first or second pregnancy	1.50	1.05 to 2.14	0.0247	1.25	0.87 to 1.78	0.2271
For women with 4+ pregnancies, where event in first, second or third pregnancy	1.65	1.09 to 2.51	0.0182	1.29	0.84 to 1.97	0.2412
Spontaneous preterm birth						
For women with 2+ pregnancies, where event in first pregnancy	0.90	0.33 to 2.43	0.84	1.0574	0.39 to 2.87	0.91
For women with 3+ pregnancies, where event in first or second pregnancy	1.14	0.56 to 2.32	0.71	1.5456	0.75 to 3.17	0.23
For women with 4+ pregnancies, where event in first, second or third pregnancy	0.40	0.10 to 1.62	0.20	0.5586	0.14 to 2.28	0.42

^a^ Adjusted for smoking, maternal age, BMI and SIMD (deprivation).

### 3.4 TFMR in Any Previous Pregnancies

Having a TFMR in any prior pregnancy was not associated with an increased risk of spontaneous preterm birth; however, due to small sample size, this result should be interpreted with caution. Women with a TFMR in any prior pregnancy were at increased risk of having a repeat TFMR.

## 4 Discussion

### 4.1 Main Findings

This study has demonstrated that having a STM significantly increases the risk of spontaneous preterm birth, repeat STM, early miscarriage, pre‐eclampsia, having a smaller baby at birth and having a baby admitted to the NNU in the pregnancy that follows. Absolute risks remain reassuringly small, but nonetheless this study significantly contributes to existing evidence that a miscarriage in the second trimester leads to an increase in adverse subsequent pregnancy outcomes. This is the first high quality comparative study to delineate the risk of spontaneous preterm birth after a STM. This study is also the first to investigate subsequent outcomes after a TFMR. The odds of having a repeat TFMR are increased in this sample, but otherwise TFMR was not associated with an increased risk of adverse perinatal risks in the next pregnancy. The results are largely reassuring for women after TFMR, though due to the small sample size the results should be interpreted with caution and our results should be confirmed in larger cohort studies.

### 4.2 Strengths and Limitations

The ability to adjust for multiple potential confounding factors is a strength of this study, but as with all observational studies there remains a risk of residual confounding for which we did not have data for. This includes a lack of data available on whether or not cervical cerclage was used in the second pregnancy, though it is unlikely the group with prior livebirth would have cerclage in the second birth, and there remained a significantly increased risk of adverse events in the women with prior STM regardless of any interventions which may have been offered in their second or subsequent pregnancies. In addition, we planned to include year of birth/miscarriage as a potential confounding variable due to historical changes in practice; however, due to missing data primarily in the STM group we could not include in the multivariate models. Thus, this is a significant limitation of this research particularly as neonatal care, obstetric surveillance and practice is likely to have changed over time though the longevity of the dataset remains a strength to enable research on subsequent pregnancy outcomes. A strength of this study was the ability to define gestation at miscarriage and TFMR which is collected within the AMND. The population studied was predominantly white women over the geographical area of Aberdeen; therefore, affecting the generalisability of the results. Cause of STM was unknown in this study and represents a significant limitation as different pathological causes may confer differing risks on subsequent pregnancy outcomes, such as cervical insufficiency versus infection; thus, this remains unanswered and future research should explore the impact of individual causes of STM, much like the calls for similar within recent preterm birth research. As an observational study, this research cannot prove causation but is a valuable addition to the previously low quality and limited evidence on pregnancy outcomes after STM and TFMR and is hypothesis‐generating. In terms of Hill's criteria for causality [25] for the associations found in this study after STM convey plausibility, consistency, strength, biological gradient and specificity. A major strength of this study is the high quality and detailed data source [20], allowing us to determine a reliable definition of spontaneous preterm birth for our primary outcome. However, we do acknowledge there is a risk someone will be coded as having had their labour induced or will have had a pre‐labour Caesarean who initially began as a PPROM. Though we are reassured that the AMND elective Caesarean coding does not include emergency Caesarean sections which included women in labour. We also note the significant limitation posed because of missing demographic details such as BMI which were much less frequently recorded for the pregnancy loss cohorts than the controls, and likely represents ‘missing not at random’ data; therefore, use of multiple imputation methods would not be appropriate. We assume this relates to the routinely collected data collection being poorer for late miscarriage and TFMR than livebirths which reflects a wider issue where data on pregnancies which end in miscarriage is less complete. Finally, generalisability of the findings to contemporary practice should be considered with appropriate caution, given major changes in prenatal diagnosis, thresholds for termination for medical reasons, and preventive strategies in subsequent pregnancies over recent times.

### 4.3 Interpretation (In Light of Other Evidence)

Our research agrees with previous studies which have demonstrated that pregnancy loss in the second trimester may confer an increased risk of repeat pregnancy loss and preterm birth [11, 26]. Having a history of early miscarriage, recurrent early miscarriages or a prior stillbirth significantly increases the risk of adverse outcomes in subsequent pregnancies, including: preterm birth, neonatal death and pre‐eclampsia [7, 8, 27, 28]. Lamont et al. [21] found that women with a previous stillbirth are almost five times more likely to have a stillbirth in subsequent pregnancy, compared with women with a previous live birth (odds ratio [OR] 4.77, 95% confidence interval [CI]: 3.70–6.15). Similarly, Wu et al. [29] found women with recurrent first trimester miscarriages are at increased risk of preterm birth (OR 1.60 [95% CI: 1.45–1.78]). Thus, our findings are plausible for adverse events after second trimester loss.

Our research on TFMR also agrees with previous research. A previous study using AMND data on all induced abortions and subsequent risk of preterm birth similarly found no increased risk [12]. A more recent study conducted in Amsterdam also revealed that having medical termination of pregnancy in the second trimester did not increase the risk of subsequent spontaneous preterm birth [30]. Women with a previous TFMR were more likely to have another TFMR but the absolute numbers remained very low and this may reflect that a proportion of women with a prior TFMR may be older or have a genetic predisposition—though this is our speculation and we did not have the data available to confirm. We note the significant difference in neonatal unit admission in our data (Table 4) but the reasons for this are not clear from our research as there were very few preterm births in the women who had a TFMR. Whilst our research is hindered by a small sample meaning there is a risk of type 2 error and an underpowered sample, women can be reassured and future research should investigate pregnancies after TFMR in larger cohorts to confirm or refute our results.

## 5 Conclusions

This study suggests that women are at increased risk of spontaneous preterm birth, repeat second trimester miscarriage, early miscarriage and pre‐eclampsia in the second pregnancy following an initial second trimester miscarriage. Where second trimester miscarriage occurs in any of the first four pregnancies the pregnancy after loss is at > 1.5 times greater risk of spontaneous preterm birth and repeat second trimester miscarriage. Women whose first pregnancy ends in TFMR are more likely to have a repeat TFMR, but otherwise we found no increased risk of spontaneous preterm birth, pre‐eclampsia, antepartum haemorrhage, early miscarriage or birthweights of subsequent pregnancies after TFMR.

Antenatal care surveillance and patient counselling should be researched and developed for women with a prior STM, who are at risk of spontaneous preterm birth and other adverse obstetric outcomes. Women after TFMR can be reassured by our findings; however, larger cohorts are needed to confirm or refute our results. Women with STM should be counselled that they are at increased risk of adverse outcomes, including spontaneous preterm birth, but reassuringly absolute incidence remains low, whilst also acknowledging that the current evidence is very limited. Our work is hypothesis generating, and large, contemporary, and ideally prospective cohort studies should continue to explore the impact of a STM or TFMR in the second trimester.

We propose that standardised clinical guidance and care planning is needed for pregnancies after second trimester pregnancy loss akin to pathways now established for pregnancies after stillbirth [31]. To date, there are no national UK or international guidelines for clinicians on second trimester pregnancy loss. It is vital that future research is aimed at understanding why the initial loss has occurred and whether these different pathological processes explain the propensity for adverse pregnancy outcomes in subsequent pregnancies. This additional understanding needs to extend to appreciate how clinicians and maternity services might intervene to reduce the risk in subsequent pregnancies of pathologies such as placental dysfunction, including interventional trials specifically for women with a prior STM.

## Author Contributions

A.M.F.W. and A.E.P.H. conceived the idea for the study and designed the study. A.M.F.W. applied for approvals. A.M.F.W., K.S., and A.E.P.H. designed and planned the statistical analyses. K.S. analysed data. A.M.F.W. wrote the first draft of the paper and edited subsequent drafts. A.E.P.H. and K.S. edited each draft of the paper and commented on the final draft.

## Funding

This research was funded by Tommy's UK, the baby and pregnancy loss charity.

## Ethics Statement

The AMND steering committee has overarching ethical approval for studies which use pseudo‐anonymised data with no data linkage and therefore formal ethics approval was not required.

## Conflicts of Interest

K.S. has no conflicts of interest to declare. A.M.F.W. holds research grants from Chief Scientist Office Scotland, NIHR EME, and Tommy's charity. A.E.P.H. has research grants from NIHR RfPB and Tommy's Charity.

## Supporting information


**Table S1:** Demographic characteristics compared between women with a second trimester miscarriage (STM) in their second, third or fourth pregnancies compared to women with livebirths (LBs) in pregnancy 1–4 as applicable (RQ2).
**Table S2:** Second pregnancy outcomes after first to fourth pregnancy events in exposed and unexposed women (RQ2).
**Table S3:** Demographic and second pregnancy characteristics of women with a first TFMR compared to women with a first livebirth.
**Table S4:** Subsequent pregnancy outcomes comparing women with and without a history of TFMR in any previous pregnancy.

## Data Availability

Research data available by application to the Aberdeen Maternity and Neonatal Databank Steering Committee (www.abdn.ac.uk/amnd).
