# Supplementary material for: Pregnancy Outcomes After Second Trimester Pregnancy Loss and Termination for Medical Reasons Before 24 Weeks: A Historical Cohort Study [PASTeL‐2]
Source: BJOG. 2026 Jan 25;133(6):1200–12. doi: 10.1111/1471-0528.70161 (PMC13040414; doi:10.1111/1471-0528.70161)
Supplement: Supplementary file 1 — Table S1: Demographic characteristics compared between women with a second trimester miscarriage (STM) in their second, third or fourth pregnancies compared to women with livebirths (LBs) in pregnancy 1–4 as applicable (RQ2). Table S2: Second pregnancy outcomes after first to fourth pregnancy events in exposed and unexposed women (RQ2). Table S3: Demographic and second pregnancy characteristics of women with a first TFMR compared to women with a first livebirth. Table S4: Subsequent pregnancy outcomes comparing women with and without a history of TFMR in any previous pregnancy. [file BJO-133-1200-s001.docx]

**Table S1 Demographic characteristics compared between women with a second trimester miscarriage (STM) in their second, third or fourth pregnancies compared to women with livebirths (LBs) in pregnancy 1-4 as applicable (RQ2)**

| **Demographic characteristic** | **Women with 2 or more recorded pregnancies** | | | **p-value** | **Women with 3 or more recorded pregnancies** | | | **p-value** | **Women with 4 or more recorded pregnancies** | | **p-value** |
| --- | --- | --- | --- | --- | --- | --- | --- | --- | --- | --- | --- |
|  | **Women with previous livebirths (N = 64495) n(%)** | **Women with previous STM (N=911) n(%)** | |  | **Women with previous livebirths**  **(N = 26162) n(%)** | **Women with previous STM (N = 1338) n(%)** | |  | **Women with previous livebirths**  **(N = 10070) n(%)** | **Women with previous STM (N=971) n(%)** |  |
| **Age**** | | | | |  | |  |  |  |  |  |
| **16-25** | 19993 (31) | | 410 (45) | <0.01* | 6017 (23) | | 375 (28) | <0.01* | 1712 (17) | 194 (20) | 0.01* |
| **25-35** | 38052 (59) | | 410 (45) |  | 16221 (62) | | 803 (60) |  | 6243 (62) | 622 (64) |  |
| **36+** | 6450 (10) | | 91(10) |  | 3924 (15) | | 160 (12) |  | 2115 (21) | 155 (16) |  |
| **BMI** | | | | |  | |  |  |  |  |  |
| **<= 20** | 5941 (9.1) | | 55 (6.0) | <0.01* | 2271 (8.7) | | 95 (7.1) | <0.01* | 878 (8.7) | 75 (7.7) | <0.01* |
| **20-25** | 30759 (47.3) | | 240 (26.3) |  | 12462 (47.6) | | 543 (40.6) |  | 4713 (46.8) | 412 (42.4) |  |
| **26-30** | 13894 (21.4) | | 134 (14.7) |  | 5948 (22.7) | | 272 (20.3) |  | 2463 (24.5) | 211 (21.7) |  |
| **>30** | 6292 (9.7) | | 75 (8.2) |  | 2846 (10.9) | | 123 (9.2) |  | 1211 (12.0) | 107 (11.0) |  |
| **Missing** | 8109 (12.5) | | 407 (44.7) |  | 2635 (10.1) | | 305 (22.8) |  | 805 (8.0) | 166 (17.1) |  |
| **Smoking status** | | | | |  | |  |  |  |  |  |
| Non smoker | 34570 (53.2) | | 412 (45.2) | <0.01* | 13907 (53.2) | | 700 (52.3) | 0.06 | 5248 (52.1) | 521 (53.7) | 0.08 |
| Ex smoker | 1627 (2.5) | | 21 (2.3) |  | 626 (2.4) | | 18 (1.3) |  | 238 (2.4) | 19 (2.0) |  |
| Smoker | 14455 (22.2) | | 256 (28.1) |  | 7160 (27.4) | | 390 (29.1) |  | 3118 (31.0) | 317 (32.6) |  |
| Missing | 14343 (22.1) | | 222 (24.4) |  | 4469 (17.1) | | 230 (17.2) |  | 1466 (14.6) | 114 (11.7) |  |
| **SIMD** | | | | |  | |  |  |  |  |  |
| 1-5 | 15053 (23.2) | | 232 (25.5) | <0.01* | 7057 (27.0) | | 351 (26.2) | 0.52 | 3123 (31.0) | 293 (30.2) | <0.01* |
| 6-10 | 30228 (46.5) | | 356 (39.1) |  | 10147 (38.8) | | 540 (40.4) |  | 3186 (31.6) | 374 (38.5) |  |
| Missing | 19714 (30.3) | | 323 (35.5) |  | 8958 (34.2) | | 447 (33.4) |  | 3761 (37.3) | 304 (31.3) |  |

****percentages only shown due to low counts in missing values.**

**Table S2 Second pregnancy outcomes after first to fourth pregnancy events in exposed and unexposed women (RQ2)**

| **Subsequent pregnancy outcome** | **Women with 3 recorded pregnancies** | | **p-value** | **Women with 4 recorded pregnancies** | | **p-value** | **Women with 5 recorded pregnancies** | | **p-value** |
| --- | --- | --- | --- | --- | --- | --- | --- | --- | --- |
|  | **Women with previous livebirths**  **(N = 64495)** | **Women with previous STM (N=911)** |  | **Women with previous livebirths**  **(N = 26162)** | **Women with previous STM (N = 1338)** |  | **Women with previous livebirths**  **(N = 10070)** | **Women with previous STM**  **(N = 971)** |  |
| **Subsequent late miscarriage** | | | |  |  |  |  |  |  |
| **No** | 63418 (97.6) | 860 (94.4) | <0.01* | 25422 (97.2) | 1283 (95.9) | 0.009* | 9766 (97.0) | 913 (94.0) | <0.01* |
| **Yes** | 1577 (2.4) | 51 (5.6) |  | 740 (2.8) | 55 (4.1) |  | 304 (3.0) | 58 (6.0) |  |
| **Subsequent first trimester miscarriage** | | | |  |  |  |  |  |  |
| **No** | 59690 (91.8) | 752 (82.5) | <0.01* | 23698 (90.6) | 1163 (86.9) | <0.01* | 8984 (89.2) | 853 (87.8) | 0.21 |
| **Yes** | 5305 (8.2) | 159 (17.5) |  | 2464 (9.4) | 175 (13.1) |  | 1086 (10.8) | 118 (12.2) |  |
| **Subsequent spontaneous preterm labour** | | | |  |  |  |  |  |  |
| No | 62263 (95.8) | 837 (91.9) | <0.01* | 24973 (95.5) | 1269 (94.8) | <0.01* | 9575 (95.1) | 909 (93.6) | <0.01* |
| Yes | 1616 (2.5) | 59 (6.5) |  | 671 (2.6) | 59 (4.4) |  | 284 (2.8) | 52 (5.4) |  |
| Missing | 1116 (1.7) | 15 (1.6) |  | 518 (2.0) | 10 (0.7) |  | 211 (2.1) | 10 (1.0) |  |
| **Subsequent adverse composite outcome (including ectopic pregnancy/molar pregnancy/induced abortion/early miscarriage/stillbirth)**  **[all compared to second pregnancy = livebirth]** | | | | | | | | | |
| No | 50466 (77.6) | 627 (68.8) | <0.01* | 17813 (68.1) | 966 (72.2) | 0.002* | 6005 (59.6) | 663 (68.3) | <0.01* |
| Yes | 14529 (22.4) | 284 (31.2) |  | 8349 (31.9) | 372 (27.8) |  | 4065 (40.4) | 308 (31.7) |  |

**Table S3 Demographic and second pregnancy characteristics of women with a first TFMR compared to women with a first livebirth**

| **Demographic characteristic** | **All women**  **(N = 65131)** | **Women with 1^st^ TFMR (N=177)** | | **Women with 1^st^ livebirth**  **(N = 64954)** | | **p-value** | | |
| --- | --- | --- | --- | --- | --- | --- | --- | --- |
| **Age** | | | | | | | | |
| **16-25** | 21931 | | 50 (28) | | 21881 (34) | | <0.01* | |
| **25-35** | 37194 | | 97 (55) | | 37097 (57) | |  | |
| **36+** | 5786 | | 30 (17) | | 5756 (9) | |  | |
| **Missing** | 43 | | 0 | | 43 (<0.1) | |  | |
| **BMI** | | | | | |  |  |  |
| **<= 20** | 5981 | | 13 (7) | | 5968 (9) | | 0.13 |  |
| **20-25** | 30713 | | 76 (43) | | 30637 (47) | |  |  |
| **26-30** | 13667 | | 43 (24) | | 13624 (21) | |  |  |
| **>30** | 6061 | | 25 (14) | | 6036 (9) | |  |  |
| **Missing** | 8532 | | 20 (11) | | 8512 (13) | |  |  |
| **Diabetes** | | | | | | | |  |
| **No** | 64164 | | 171 (97) | | 63993 (99) | | 0.02 |  |
| **Yes** | 790 | | 6 (3) | | 784 (1) | |  |  |
| **Smoking status** | | | | | | | |  |
| Non smoker | 34063 | | 137 (77) | | 33926 (52) | | <0.01* |  |
| Ex smoker | 1709 | | 7 (4) | | 1702 (3) | |  |  |
| Smoker | 14424 | | 33 (19) | | 14391 (22) | |  |  |
| Missing | 14935 | | 0 | | 14935 | |  |  |
| **SIMD** | | | | | | | |  |
| 1-5 | 15096 | | 56 (32) | | 15043 (23) | | <0.01* |  |
| 6-10 | 30003 | | 121 (68) | | 29882 (46) | |  |  |
| Missing | 19855 | | 0 | | 19852 (31) | |  |  |
| **Maternal pre-existing hypertension** | | | | | | | |  |
| No | 56456 | | 146 (82) | | 56310 (87) | | 0.10 |  |
| Yes | 8498 | | 31 (18) | | 8467 (13) | |  |  |
| **Gestation at second birth** | | | | | | | |  |
| 0-23 | 6554 | | 28 (16) | | 6526 (10) | | 0.02* |  |
| 24-36 | 1335 | | 6 (3) | | 1329 (2) | |  |  |
| 37+ | 49771 | | 127 (72) | | 49644 (76) | |  |  |
| Missing | 7471 | | 28 (9) | | 7455 (11) | |  |  |
| **Pre-eclampsia** | | | | | | | |  |
| No | 56488 | | 146 (82) | | 56342 (87) | | 0.10 |  |
| Yes | 8466 | | 31 (18) | | 8435 (13) | |  |  |

**Table S4 Subsequent pregnancy outcomes comparing women with and without a history of TFMR in any previous pregnancy**

| **Subsequent pregnancy outcome** | **Women with two recorded pregnancies** | |  | **Women with three recorded pregnancies** | |  | **Women with four recorded pregnancies** | |  |
| --- | --- | --- | --- | --- | --- | --- | --- | --- | --- |
|  | **Women previous livebirths**  **(N = 64777)** | **Women previous TFMR (N=177)** | **p-value** | **Women with previous livebirths**  **(N = 26464)** | **Women with previous TFMR (N = 268)** | **p-value** | **Women with previous livebirths**  **(N = 10423 )** | **Women with previous TFMR**  **(N = 164)** | **p-value** |
| **Subsequent TFMR** | | | |  |  |  |  |  |  |
| **No** | 64408 (99.4) | 168 (94.9) | <0.01 | 26313 (99.4) | 260 (97.0) | <0.01 | 10359 (99.4) | 159 (97.0) | 0.004 |
| **Yes** | 369 (0.6) | 9 (5.1) |  | 151 (0.6) | 8 (3.0) |  | 64 (0.6) | 5 (3.0) |  |
| **Subsequent first trimester miscarriage** | | | |  |  |  |  |  |  |
| **No** | 59518 (91.9) | 151 (85.3) | 0.002 | 23982 (90.6) | 232 (86.6) | 0.024 | 9313 (89.4) | 137 (83.5) | 0.018 |
| **Yes** | 5259 (8.1) | 26 (14.7) |  | 2482 (9.4) | 36 (13.4) |  | 1110 (10.6) | 27 (16.5) |  |
| **Spontaneous preterm birth** | | | | | | | | | |
| **No** | ** | ** | 0.208 | 25264 (95.5) | 260 (97.0) | 0.064 | ** | ** | 0.0701 |
| **yes** | ** | ** |  | 680 (2.6) | 8 (3.0) |  | ** | ** |  |

**** not shown due to low counts**
